# The surge of RSV and other respiratory viruses among children during the second COVID-19 pandemic winter season

**DOI:** 10.3389/fped.2023.1112150

**Published:** 2023-02-01

**Authors:** Angela Riepl, Lena Straßmayr, Peter Voitl, Paulina Ehlmaier, Julian J. M. Voitl, Klara Langer, Ulrike Kuzio, Alexandra Mühl-Riegler, Bernhard Mühl, Susanne C. Diesner-Treiber

**Affiliations:** ^1^ First Vienna Pediatric Medical Center, Vienna, Austria; ^2^ Sigmund Freud University Vienna, Vienna, Austria; ^3^ Vienna Medical Innovation Center, Vienna, Austria

**Keywords:** RSV, children, acute respiratory infection, metapneumovirus, non-pharmaceutical measures, COVID-19

## Abstract

**Background:**

The non-pharmaceutical measures in the first Covid-19 winter season significantly impacted respiratory pathogens such as RSV, influenza, or metapneumovirus, which cause respiratory infections, especially in infants and young children. This longitudinal prospective study aimed to determine how less strict measures affect the pathogen profile in the second winter season.

**Methods:**

From September 2021 till the end of March 2022, 678 children (0–36 months) admitted to Vienna's largest pediatric center with an acute respiratory infection were enrolled in this study. The researchers performed nasal swabs and tested them by multiplex PCR for 23 respiratory pathogens, chronicled clinical features and treatment, and analyzed the effect of lockdown on the pathogen prevalence.

**Results:**

The 815 smears of 678 children revealed the most common pathogens to be rhino-/enterovirus (38.5%), RSV (26.7%), and metapneumovirus (7.2%). The lockdown interrupted the early RSV onset in September [RR 0.367, CI (0.184–0.767), *p* = 0.003], while no effects on the other pathogens were found. Metapneumovirus started circulating in January. Influenza was only sporadically detected. The hospitalization rate was significantly higher than last season due to RSV [OR 4.089, 95%CI (1.414–11.827), p-adj = 0.05].

**Conclusion:**

With more flexible non-pharmaceutical measures, children aged 0–36 months started presenting again with viral pathogens, such as RSV and metapneumovirus. RSV, associated with a high hospitalization rate, had a very early onset with an abrupt interruption due to the only lockdown.

## Introduction

Respiratory infections in children occur several times a year (1). The responsible pathogens are the respiratory syncytial virus (RSV), rhinovirus, influenza virus, human coronavirus, adenovirus, parainfluenza virus, and metapneumovirus. Respiratory viral infections are the leading cause of hospitalization in infants and young children and the second most common cause of infant mortality (2). The pathogens mentioned above can cause upper and lower respiratory tract infections with indistinguishable symptoms (2–4).

Before the Covid-19 pandemic, RSV, influenza, and human coronavirus followed a certain seasonality, typically starting between November and December in the northern hemisphere (5–7). Since the emergence of SARS CoV2, drastic measures have been adopted worldwide to prevent the virus from spreading. There were recommendations for lockdowns, social distancing, hand hygiene, and mouth and nose protection, which curbed the Covid-19 pandemic and the occurrence of other respiratory pathogens. In a longitudinal study we conducted in the first Covid-19 winter season (2020/2021), we were able to determine the absence of RSV, metapneumovirus, or influenza in a pediatric cohort with acute respiratory infections (0–24 months). In contrast, rhinoviruses, adenoviruses, and human coronaviruses continued to appear and were in part influenced by the Covid-19 measures (lockdown) (8). Similar results have been confirmed in other countries and by the Surveillance Network data (9–12). German children were spared from influenza, metapneumovirus and RSV, whereas rhino/enterovirus and adenovirus were still circulating through the pandemic (11). According to Australian and English data, a dramatic impact of the Covid-19 measures was observed for influenza and RSV (10, 12). However, the RSV activity increased after removal of local Covid-19 restrictions and after relaxation of Australian state borders (10).

Based on these observations, after an RSV-free season and the related lack of protective immunity, we expected the following seasonal epidemic to be more aggressive, with an earlier outbreak than before Covid-19. Therefore, this prospective longitudinal, monocentric study aimed to investigate how respiratory infections, especially RSV, develop in children up to three years of age during the second Covid-19 winter season, with eased preventive measures, and whether the pathogen profile has evolved. In specific, we focused on the epidemiological profile, the clinical characteristics and disease severity, indicated by hospitalization rate. As children are a major cause for viral spreading, especially of influenza or RSV, it is important to understand the direct and long-lasting impact of pandemic measures and whether children are at risk for respiratory infections with atypical seasonality or higher disease severity as soon as the preventive measures are lifted.

## Materials and methods

### Study design

The design of this prospective, longitudinal study remains unvaried to our recent publication (8) including patients from the primary health care setting, except for the following changes: inclusion of children 0–36 months, recruitment phase September 2021 to March 2022, multiplex PCR including SARS CoV2.

This prospective longitudinal study was conducted at the primary health care center, “First Vienna Pediatric Medical Center,” in the second winter season during the Covid-19 pandemic in Austria.

Healthy children (0–36 months) who presented with an acute respiratory infection (no longer than ten days) exhibiting at least one common cold symptom, such as sore throat, cough, rhinitis, nasal congestion, or otitis media, with or without fever were actively invited to participate in this study. Recruitment was done during the regular doctor's appointments from Monday to Friday during the recruitment period. Exclusion criteria were: premature infants (<37 weeks gestation) and infants with severe congenital or chronic diseases (e.g., severe congenital pulmonary or neuromuscular diseases, severe immunodeficiency, chromosomal abnormalities, and cardiac malformations).

The Ethics Committee of the Medical University of Vienna approved (EK-No. 1864/2020) this study. Parents signed the declaration of consent after being informed about the aims and procedure of our research.

The family's socioeconomic status (parents' education, living situation, marital status, number of siblings), the patient's general clinical data (weight, height, calculated BMI, chronic diseases), the clinical features of the acute respiratory infection (rhinitis, nasal congestion, fever, cough, pharyngitis), and treatment recommendations (decongestant nasal spray, hypertonic saline spray, analgesics, antibiotics, inhalation therapy [salbutamol as short-acting betamimetics (SABA), inhaled corticosteroids (ICS)], inhalation with sodium chloride), systemic corticosteroids, and indications for hospitalization, were documented in a case report form.

### Study procedure

A trained study team collected anterior nose swabs. The samples were stored in 0.9% sodium chloride solutions until further analysis. Tests were conducted using the multiplex PCR respiratory panel (Biofire FilmArray Respiratory panel 2.1 plus kit) for adenovirus, human coronaviruses (229E, HKU1, OC43, NL63), SARS CoV2, metapneumovirus, rhinovirus/enterovirus, influenza A (H1, H1-2009, H3), influenza B, parainfluenza virus 1–4, RSV, MERS-CoV, Chlamydia pneumoniae, Mycoplasma pneumoniae, Bordetella pertussis, and Bordetella parapertussis. According to the manufacturer, sensitivity and specificity for nasopharyngeal swabs are high (13). The Ethics Committee, however, required a less invasive nasal swab.

In the case of recurrent acute infections in one child, nasal swabs were repeated only with healthy periods in between the visits of at least one week. However, sociodemographic and patient-specific data were collected only at the first visit.

### COVID-19 regulations

Covid-19 restrictions were less stringent than in the past year. The Austrian Covid-19 regulations were constantly monitored on the website of the Federal Ministry of Social Affairs, Health, Care and Consumer Protection (14).

In winter 2021, the many rules for controlling the spread of the SARS CoV2 pathogen included general recommendations such as keeping distance, hand hygiene, and wearing FFP2 masks or mouth and nose protection in public spaces and schools from the sixth year of life. In Austria, Covid vaccination has been recommended to everyone older than 12 years since May 2021 and has been available for children above five since November 25, 2021. Everyone was imposed a general lockdown from November 22, 2021 to December 12, 2021. There was an all-day curfew, and shops, restaurants, and other venues were closed. However, schools remained open. Attendance was not mandatory for kindergartens; however, childcare options were available for parents who could not look after their children at home (14).

### Statistics

Raw data were sorted in Google Sheets and Microsoft Excel using IBM SPSS-Statistics version 27. The statistical significance level was set at *p* < 0.05.

Almost all data were categorized as nominal variables, except for the number of siblings (ordinal) and the metric variables: age, height, weight, and BMI. In addition, six age groups of 6 months each were formed: 0–6, 6–12, 12–18, 18–24, 24–30, and 30–36 months.

All individual positive results of detected pathogens were transformed into new variables, and the pathogen families were merged into broader groups without multiple infections: RSV, adenovirus, metapneumovirus, rhinovirus, coronavirus (coronaviruses 229E, HKU1, OC43, NL63), SARS CoV2, influenza (A and B), parainfluenza virus (1–4). Multiple infections (co-infections with more than one pathogen) and results without a pathogen each form a separate category.

Patient characteristics, symptoms, drugs, and pathogens, as well as the comparison of occurring symptoms or recommended therapy with existing pathogens were presented with descriptive statistics through absolute and relative frequency, depending on therapy/symptoms and on positively tested of a pathogen group.

For metric variables the mean, standard deviation, median, and first and third quartiles are reported for the number of valid values (*n*) in each case.

The positivity rate (%) of a positive group/all tested subjects in the calendar weeks of the winter semester 2020/21 (8) and winter semester 2021/22 was presented in a bar chart.

The Chi-square test (X2) tested nominal variables such as symptoms, treatment, and pathogen groups for statistically significant associations.

Associations between two categorical variables (comparison of symptoms and pathogens, as well as treatment and pathogens) are analyzed using Cramér's V, which was interpreted using the Rea and Parker's classification (*r* = 0.10–0.20 weak, *r* = 0.20–0.40 moderate, and *r* = 0.40–0.60 relatively strong association) (15).

A multiple logistic regression model tested the pathogen frequency under specific influences [independent variables: Lockdown, age (in groups = ordinal), hospitalization and siblings]. The nominal variable “*pathogen category*” represented the dependent variable and the negative sample represented the reference variable. The odds ratio (OR) and 95% confidence interval (CI) were calculated.

To calculate the effect of the lockdown on the pathogen frequency, we compared the period of the two weeks before the start of the lockdown with the two weeks after the start of one lockdown week and calculated the rate ratio (RR), and presented it with a 95% CI. We left out the first week of lockdown in the comparison since we expected an effect overlap to bias the results. After one week of lockdown, the results differentiate the interval from pre-lockdown times.

In order to avoid the risk of a higher frequency of errors due to multiple testing, the Bonferroni correction was applied.

## Results

### Characteristics of the study population

The study included 678 children [median age 16.36 months (Q1–Q3: 8–25 months), 54.6% male, 45.3% female, 0.1% missing] with acute respiratory infection from the beginning of September 2021 until the end of March 2022. In total, 815 nasal swabs were collected. Five-hundred-sixty-seven children had one swab, 85 children had two swabs, 22 children had three swabs, and three children had four swabs. All sociodemographic and general clinical characteristics are summarized in Table 1.

**Table 1:** Characterization of study population.

Age	Months	16,36 (8–25)
BMI	Kg/m2	16.21 (14.9–17.4)
Sex	Male	370 (55)
	Female	307 (45)
	Missing data	1 (<1)
Siblings	Number	0.79 (0–1)
	Missing data	3 (<1)
Housing	Community housing	55 (8)
	Apartment	505 (75)
	House	116 (17)
	Missing data	2 (<1)
Parents’ marital status	In relation	618 (91)
	Separated	56 (8)
	Missing data	4 (<1)
Highest education mother	Compulsory education	75 (11)
	Apprenticeship	193 (29)
	High school diploma	141 (21)
	Academic degree	262 (39)
	Missing data	7 (1)
Highest education father	Compulsory education	60 (9)
	Apprenticeship	260 (38)
	High school diploma	122 (18)
	Academic degree	223 (33)
	Missing data	13 (2)
Chronic diseases	Yes	16 (2)
	No	660 (97)
	Missing data	2 (<1)

Metric data are presented as median and (Q1–Q3), qualitiative values as absolute numbers and relative frequencies in percentage (%). *N* = 678.

The most common clinical symptom was cough (79.5%), followed by rhinitis (70.6%) (see Table 2). In this winter season, symptomatic treatment of children entailed decongestant nasal sprays/nasal drops (70.6%), analgesics (62.6%), and antibiotics (8.1%). Short-acting betamimetics (13%) and systemic corticosteroids (8%) were prescribed more frequently. Hospitalization was indicated in fourteen cases (1.7%) (Table 2) of which seven were RSV single infections, one RSV/rhinovirus coinfection, one RSV/rhinovirus/parainfluenza virus coinfection, two rhinovirus single infections, one metapneumovirus/rhinovirus co-infection and in two cases no pathogen was detected by multiplex PCR. Hospitalization significantly correlated with RSV detection [OR 4.089, 95%CI (1.414–11.827), p-adj = 0.05] (Supplementary Table S1).

**Table 2:** Clinical characterization and medication.

**Clinical characteristics**	
Cough	648 (80)
Rhinitis	575 (71)
Fever	400 (49)
Nasal congestion	160 (20)
Pharyngitis	150 (18)
**Prescribed medication**	
Decongestant and hypertonic saline nose spray	575 (71)
Analgesics	510 (63)
Inhalation SABA	106 (13)
Antibiotics	66 (8)
Systemic corticosteroids	65 (8)
Inhaled corticosteroids	15 (2)
0.9% sodium chloride inhalation	8 (1)
Hospitalization	14 (2)

Data are presented as absolute number and relative frequency (%), qualitative data as median (Q1–Q3).

Children had multiple symptoms and were prescribed multiple medications; therefore, multiple responses are possible. *N* = 815.

SABA, short acting beta mimetics.

### Detection of pathogens throughout the winter season

The Multiplex PCR showed no pathogen in 177 out of the 815 samples (21.8%); co-detections occurred in 108 samples (13.3%).

**Figure 1:**
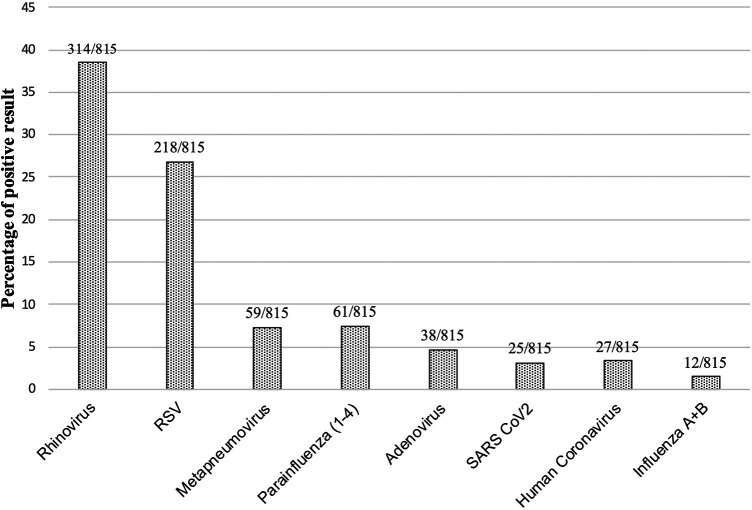
Percentage of positive pathogen result. Total numbers and percentages of positive results over the complete winter season are shown. Total number of nasal swabs: 815.

The most common pathogen was rhinovirus (*n* = 314/815, 39%), followed by RSV (*n* = 218/815, 27%), metapneumovirus (*n* = 59/815, 7%), parainfluenza viruses (1–4) (*n* = 61/815, 7%), adenovirus (*n* = 38/815, 5%), and SARS CoV2 (*n* = 25/8,153%) (Figure 1). The influenza virus was sporadic. The other tested pathogens, such as Bordetella pertussis, were not detectable throughout the winter season.

As expected, rhinovirus was associated with rhinitis and cough,. RSV led to rhinitis and fever and showed moderate but not significant association with cough (Cramér's V 0.201, *p* < 0.001, p-adj 0.5, (Supplementary Table S2). Cough was present in all metapneumovirus and parainfluenza virus-positive patients. SARS CoV2 often led to cough and fever, while adenovirus frequently associated with fever (Table 3).

**Table 3:** Frequency of symptoms and treatment per pathogen category.

	Symptoms					Treatment						
	Cough	Rhinitis	Nasal congestion	Fever	Pharyngitis	Nasal spray	Antibiotics	Analgesics	Short-acting agonists	Inhaled cortison	Systemic cortison	0.9% NaCl inhalation
Rhinovirus*	170 (73.9)	185 (80.4)	42 (18.3)	85 (37.0)	44 (19.1)	180 (78.3)	11 (4.8)	136 (59.1)	13 (5.7)	5 (2.2)	17 (7.4)	4 (1.7)
RSV*	157 (95.7)	125 (76.2)	36 (22.0)	93 (56.7)	32 (19.5)	118 (72.0)	16 (9.8)	108 (65.9)	39 (23.8)	0 (0.0)	14 (8.5)	1 (0.6)
Metapeumovirus*	47 (100)	28 (59.6)	7 (14.9)	29 (61.7)	8 (17.0)	32 (68.0)	3 (6.4)	34 (72.3)	15 (31.9)	2 (4.3)	3 (6.4)	0 (0.0)
Parainfluenza*	36 (100)	27 (75)	5 (13.9)	17 (47.2)	7 (19.4)	23 (64.0)	5 (13.9)	21 (58.3)	4 (11.1)	0 (0.0)	3 (8.3)	2 (5.6)
Adenovirus*	4 (30.8)	3 (23.1)	2 (15.4)	10 (76.9)	4 (30.8)	5 (38.5)	2 (15.4)	10 (76.9)	2 (15.4)	0 (0.0)	1 (7.7)	0 (0.0)
SARS CoV2*	15 (65.2)	10 (43.5)	4 (17.4)	18 (78.3)	3 (13.0)	14 (60.8)	1 (4.3)	18 (78.3)	2 (8.7)	2 (8.7)	4 (17.4)	0 (0.0)
Human Coronavirus*	9 (64.3)	9 (64.3)	5 (35.7)	7 (50)	2 (14.3)	9 (64,2)	1 (7.1)	6 (42.9)	1 (7.1)	1 (7.1)	0 (0.0)	0 (0.0)
Influenza A + B*	1 (100)	0 (0)	1 (100)	0 (0)	0 (0)	0 (0.0)	0 (0.0)	0 (0.0)	0 (0.0)	0 (0.0)	1 (100)	0 (0.0)
Multiple infections	92 (85.2)	85 (78.7)	12 (11.1)	53 (49.1)	18 (16.7)	75 (69.5)	15 (13.9)	65 (60.2)	21 (19.4)	2 (1.9)	9 (8.3)	0 (0.0)
Negative	116 (65.5)	102 (57.6)	46 (26)	88 (49.7)	31 (17.5)	118 (66.6)	12 (6.8)	111 (62.7)	9 (5.1)	3 (1.7)	13 (7.3)	1 (0.6)
Total	647	574	160	400	149	574	66	509	106	15	65	6

Data are presented as absolute numbers and relative frequencies (%) of positive tested pathogen group.

* Indicates numbers of single infections without co-infections.

The prescribed treatment per pathogen group is shown in Table 3 and Supplementary Table S3. Twenty-four percent of RSV cases and 32% of metapneumovirus cases were treated with beta-2 agonists due to signs of airway obstruction according to the physician's documentation.

Next, we investigated whether specific age groups or the presence of siblings altered the risk of contracting a pathogen. We found a significantly lower risk for rhinoviruses (OR 0.517, 95% CI 0.357–0.749) in the age group 6–12 months after correction for multiple testing (p-adj =0.009), but a higher risk of co-infections (OR 3.157, 95% CI 1.499–6.63), p-adj = 0.018). There were no significant incidences in the 12–18, 18–24 months, and 30–36 months age groups, while there was a significantly higher risk of rhinovirus infection in the 24–30 months [OR 2.10, 95% CI (1.291–3.420), *p*-adj = 0.027] (Supplementary Table S4).

Having siblings significantly increases the incidence of rhinovirus (OR 1.686, 95% CI 1.240–2.293), p-adj = 0.009) while the risk of metapneumovirus decreases, which, however, was not significant after Bonferroni correction (OR 0.445, 95% CI 0.236–0.875), *p* = 0.018, p-adj = 0.162) (Supplementary Table S5).

### Longitudinal analysis of infectious pathogens over the winter months

**Figure 2:**
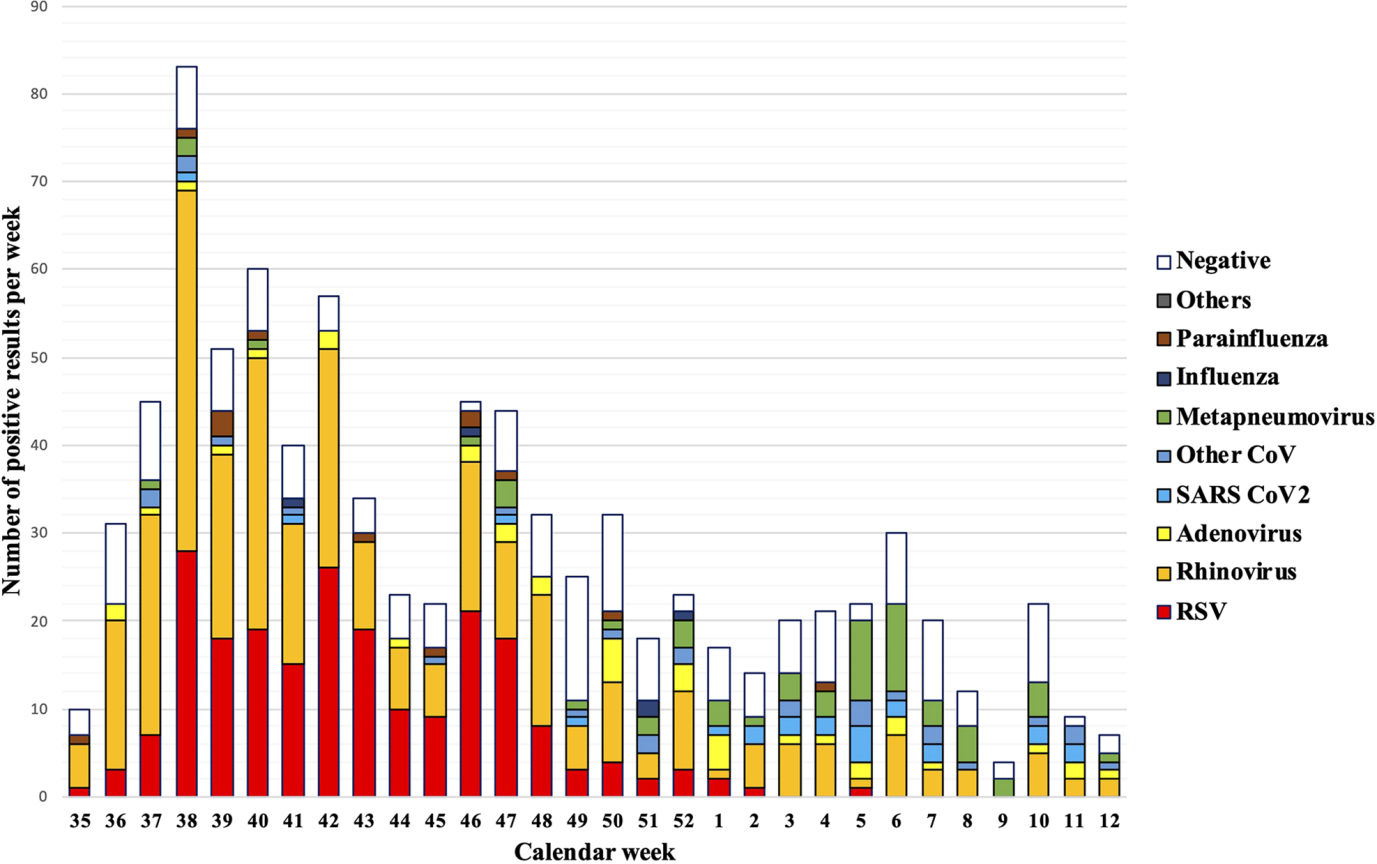
Pathogen detection rates per calendar week. A full lockdown was ordered throughout Austria in calendar weeks 47–49.

Infections with multiple viruses and single infections with rhinovirus frequently occurred throughout the entire winter season. For example, in September, RSV was already circulating, with in total 164 RSV cases without co-infections and 218 single RSV cases and RSV co-occurring with other pathogens) over the winter season. However, RSV decreased steadily in November and December, with only sporadic cases at the beginning of 2022. In contrast, infections with metapneumoviruses peaked in the first weeks of 2022. All other pathogens were occasionally found without any significant seasonal profile (Figure 2).

### Effect of COVID-19 lockdown on the pathogenic profile

**Figure 3:**
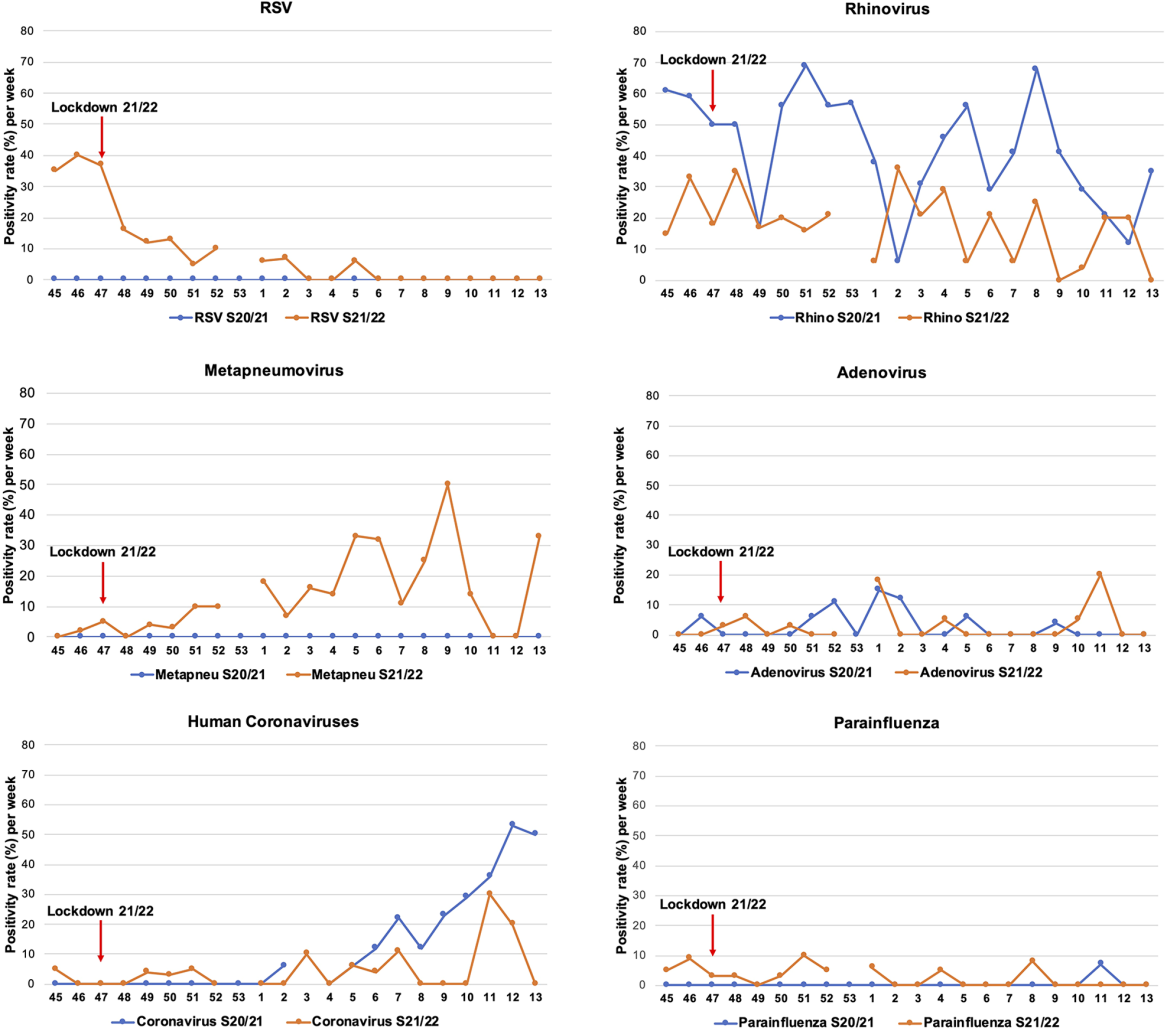
Positivity rate of pathogens in both COVID-19 winter seasons. The positivity rate of pathogens in the winter season 2020/2021 (blue lines) and 2021/2022 (orange lines) are presented. The lockdown in the winter season 2021/2022 starting from calendar week 47 is shown (red arrow).

In contrast to the previous winter season, only one Covid-19 lockdown was ordered in calendar weeks 47–49. The lockdown significantly reduced the risk for RSV, which showed a decrease in the lockdown calendar weeks 47/48/49 (62.4% risk reduction, RR 0.376, 95% CI (0.184–0.767, *p* = 0.003, p-adj = 0.021). All other pathogens were not affected by the Covid-19 measures this season (Figure 3 and Table 4).

**Table 4:** Effect of lockdown on the pathogen groups.

	RR	95% CI	*p*-value	p-adj.
RSV	0.376	0.184–0.767	0.003	0.021
Adenovirus	–	–	0.130	0.91
Metapneumovirus	1.127	0.072–17.544	0.932	>0.999
Rhinovirus	0.995	0.550–1.798	0.986	>0.999
Influenza	–	–	–	
Coronaviruses	1,127	0.072–17.544	0.932	>0.999
Parainfluenzavirus	0.222	0.027–1.873	0.126	0.882
Multiple infections	0.705	0.245–2.028	0.513	>0.999

Rate ratio (RR), 95% CI, *p*-value and p-adj values are given.

When we compared the positivity rate to the major pathogens between the two winter seasons (8), a lower rhinovirus and human coronaviruses detection rate emerged in the more recent winter. While RSV and metapneumovirus predominantly circulated, parainfluenza viruses decreased in 2021/2022. Adenovirus infections remained unvaried (Figure 3).

## Discussion

This prospective longitudinal cohort study analyzed the epidemiological profile of respiratory viruses in children with acute respiratory infections during the second Covid-19 pandemic winter season (2021/2022). Children (0–36 months) presenting to Austria's largest primary health care center with symptoms of respiratory infections tested positive mainly for rhinovirus, RSV, and metapneumovirus.

As in the previous season, influenza was only sporadically diagnosed and might not display an influenza infection but rather be caused by viral shedding after live-attenuated nasal influenza vaccination, which was done one week prior to detection (16). RSV was detectable early in September, while metapneumovirus only peaked in January.

The only lockdown (weeks 47–49) led to a 62.4% reduction in RSV cases, with sporadic evidence during the rest of the season. Compared to the 2020/2021 winter season, the different pathogen profiles in 2021/2022 determined a significantly higher positivity rate for RSV and metapneumovirus, while rhinoviruses and human coronaviruses were less frequently detectable. A comparison for SARS CoV2 between the winter seasons is unfeasible as no testing was conducted in the first season.

The general comparison of the two winter seasons revealed the difference in the pathogen profile, symptoms, and treatment. In 2021/2022, parents reported cough as the most frequent symptom, followed by rhinitis, as opposed to the previous winter season, when rhinitis surpassed cough. This could relate to the rhinovirus infection being less diffused in the 2021/2022 season. In general, the symptoms were indistinguishable between the infections. However, RSV showed a moderate association with cough and infections with either parainfluenza virus or metapneumovirus led always to cough. Treatment regimens were comparable, with SABA inhalation being prescribed more frequently in 2021/2022 (13% vs. 5%), especially for metapneumovirus and RSV infections. On the one hand, patients with RSV and metapneumovirus suffered more frequently from cough, on the other hand airway obstruction were reported by the physicians more frequently.

The hospitalization rate increased by 1.7% compared to the previous year and correlated significantly with RSV infection [OR 4.089, 95% CI (1.414–11.827), p-adj = 0.05]. Our data confirm reports that non-pharmaceutical interventions during the Covid-19 pandemic diminished the hospitalization rate for children with other viral respiratory pathogens (17).

This winter season, we also decided to evaluate 36-month-olds, as the pandemic “immunity gap” might determine a surge in RSV, metapneumovirus, and influenza infections among very young children. We excluded children with chronic heart or lung diseases, severe congenital diseases as this group is more likely to become seriously ill with respiratory viruses such as RSV (18). In addition, vulnerable populations are usually protected against RSV in advance with passive immunization (18, 19), are typically admitted directly to the hospital, and may have a different risk profile for infectious diseases compared to healthy children.

Regrettably, the lack of comparable studies in Austria conducted before Covid-19 prevents comparisons with our cohort. However, our data and national and international surveillance data reveal a very early RSV season with a peak in September/October (6). A German surveillance study showed the first isolated cases of RSV among children aged between 0 and 4 as early as July 2021, with a significant increase in RSV infections in October 2021 (20). Covid-19, RSV, and parainfluenza epidemic waves in children were also observed in a Finnish surveillance study after restrictions were lifted in September 2021. RSV was noticed earlier than usual (21). Non-European data also confirmed the trend of an atypical season (22).

The effect of the unpredicted RSV season on the degree of severity remains controversial, with some data showing comparable severity (23) and others reporting worse clinical pictures among young children (24). Our results showed that compared to the first Covid-19 winter season, the children required more hospitalization, which was associated with the detection of RSV. However, we cannot conclude that the RSV infections were more severe than before Covid-19. A study from Germany confirmed a higher RSV hospitalization rate than in previous years and an increase in RSV infection rates in 2- to 4-year-old children (25).

Interestingly, our study could not find evidence of an influenza wave during the observation period from September 2021 to March 2022. According to the national surveillance data, however, in the weeks that followed, outside our study recruitment period, influenza activity was evident, which was also abnormally late compared to the pre-Covid-19 years (26).

The fact that non-pharmaceutical measures have an effect on respiratory viruses of varying degrees is undisputed and widely published (12, 27, 28).

In our study, too, the connection between the RSV decline and the Covid-19 lockdown measures is unmistakable, but the causality cannot be fully demonstrated. It can also be hypothesized that this is a natural progression of the RSV season, which ends prematurely due to the early onset. However, the Austrian data on the RSV seasons before Covid-19 prove a significantly prolonged season, regardless of its beginning (6). It can therefore be assumed that the lockdown directly impacted the course of RSV in the 2021/2022 winter season.

The lockdown did not appear to affect other pathogens' cycles. The late metapneumovirus detection rate in the early weeks of 2022 reflects pre-Covid-19 patterns, as shown by other international data (29).

Our results corroborate the hypothesis that children and infants were only indirectly affected by non-pharmaceutical measures. Kindergartens and schools had higher attendance in the 2021/22 winter season, which means that older children often transmit respiratory infections to their younger siblings at home (30, 31). As for rhinoviruses, the presence of siblings increases the risk of infection by a factor of 1.68. The different impact of non-pharmaceutical measures on respiratory pathogens remains open and needs further investigation.

## Limitations

Although our patients come from the entire Vienna region, the monocentric design is a limiting factor and might not be representative for other settings. Presumably, certain population groups are more likely to reach the hospital as the first point of contact or to stay home to observe precautionary measures (14), which would explain the 3% SARS CoV2 positivity rate and constitute a bias. The hospitalization rate might not be representative for all acute ill children as more severe cases might have directly consulted the hospital than making an appointment in the primary health care centre as first point.

Although many parents very well received the study, we were unable to determine how many declined to participate. Families with university degrees were more likely to accept, representing a bias. Nevertheless, the comparability of our data with the national surveillance data makes it representative.

The nasal swab was preferred to the nasopharyngeal sampling. Although a possible bias, the less invasive test yields valid results also described in the literature (32–35).

## Conclusion

Conversely to what happened last year, loose non-pharmaceutical measures triggered a wave of viral pathogens among children aged 0–36 months. The lockdown threw off the RSV seasonality but had a minor or no impact on the cycle of other pathogens. The pathogen profile with recurrent metapneumovirus and parainfluenza virus returned to pre Covid-19 times patterns, while influenza did not follow its regular season. Therefore, research efforts should examine the direct effects of non-pharmaceutical actions on the spread and seasonality of respiratory pathogens.

## Data Availability

The raw data supporting the conclusions of this article will be made available by the authors, without undue reservation.
