# Supplementary material for: The surge of RSV and other respiratory viruses among children during the second COVID-19 pandemic winter season
Source: Front Pediatr. 2023 Feb 1;11:1112150. doi: 10.3389/fped.2023.1112150 (PMC9929140; doi:10.3389/fped.2023.1112150)
Supplement: Supplementary file 1 [file Supplementarytables.docx]

**Supplementary files**

| **Supplementary table 1. Risk for hospitalization per pathogen group** | | | |
| --- | --- | --- | --- |
| **Pathogen** | **OR** | **95% CI** | **p-value / p-adjust** |
| RSV | 4.089 | 1.414 – 11.827 | **0.005 / 0.05** |
| Rhinovirus | 0.417 | 0.093 – 1.880 | 0.241 / >0.999 |
| Multiple infections | 1.803 | 0.495 – 6.568 | 0.365 / >0.999 |
| Negative | 0.594 | 0.132 – 2.680 | 0.494 / >0.999 |
| OR: Odds ratio, p-adjust: p-value after Bonferroni correction. Only results of hospitalized patients are depicted. | | | |

| **Supplementary table 2. Association of pathogen group and symptoms** | | | | | |
| --- | --- | --- | --- | --- | --- |
|  | Cough | Rhinitis | Blocked nose | Fever | Pharyngitis |
| RSV | **<0.001** / 0.5 / 0.201 | 0.077 / >0.999/ 0.062 | 0.413 / >0.999 / 0.029 | **0.031** / >0.999 / 0.075 | 0.661 / >0.999 / 0.015 |
| Adenovirus | **<0.001** / 0.5 / 0.154 | **<0.001** / 0.5 / 0.133 | 0.695 / >0.999 / 0.014 | **0.044** / >0.999 / 0.071 | 0.242 / >0.999 / 0.041 |
| Metapneumovirus | **<0.001** / 0.5 / 0.125 | 0.087 / >0.999 / 0.060 | 0.395 / >0.999 / 0.030 | 0.077 / >0.999 / 0.062 | 0.812 / >0.999 / 0.008 |
| Rhinovirus | **0.012** / 0.6 / 0.088 | **<0.001** / 0.5/ 0.136 | 0.523 / >0.999 / 0.022 | **<0.001** / 0.5 /0.154 | 0.710 / >0.999 / 0.013 |
| SARS CoV2 | 0.083 / >0.999 / 0.061 | **0.004** / 0.2 / 0.102 | 0.779 / >0.999 / 0.010 | **0.005** / 0.25/ 0.099 | 0.506 / >0.999 /0.023 |
| Influenza virus | 0.612 / >0.999 / 0.018 | 0.121 / >0.999 / 0.054 | 0.043 / >0.999 / 0.071 | 0.325 / >0.999 / 0.035 | 0.636 / >0.999 / 0.017 |
| Human coronavirus | 0.152 / >0.999 /0.050 | 0.601 / >0.999 / 0.018 | 0.128 / >0.999 / 0.053 | 0.952 / >0.999 / 0.002 | 0.693 / >0.999 / 0.014 |
| Parainfluenza virus | **0.002** / 0.1 / 0.109 | 0.554 / >0.999 / 0.021 | 0.371 / >0.999 / 0.031 | 0.808 / >0.999 / 0.009 | 0.859 / >0.999 / 0.006 |
| Multiple Infections | 0.121 / >0.999 / 0.054 | **0.047** / >0.999 / 0.070 | **0.016** / 0.8 / 0.084 | 0.977 />0.999 / 0.001 | 0.632 / >0.999 / 0.017 |
| Negative | **<0.001** / 0.05 / 0.184 | **<0.001** / 0.5 /0.150 | **0.017** / 0.85 / 0.084 | 0.876 / >0.999 / 0.005 | 0.752 / >0.999 / 0.011 |
| p-value / adj.p-value/Cramer’s V | | | | | |

| **Supplementary table 3. Association of pathogen group and treatment** | | | | | | | |
| --- | --- | --- | --- | --- | --- | --- | --- |
|  | **Decongestant/**  **Hypertonic nose spray** | **Antibiotics** | **Analgesics** | **Inhalation SABA** | **Inhaled corticosteroids** | **Systemic corticosteroids** | **0.9% sodium chlorid inhalation** |
| RSV | 0.343 / >0.999 / 0.064 | 0.390 / >0.999 / 0.030 | 0.336 / >0.999 / 0.034 | <0.001 / 0.07 / 0.160 | 0.049 / >0.999 / 0.069 | 0.775 / >0.999 / 0.010 | 0.587 />0.999 / 0.019 |
| Adenovirus | 0.054 / >0.999 / 0.097 | 0.334 / >0.999 / 0.034 | 0.282 / >0.999 / 0.038 | 0.800 / >0.999 / 0.009 | 0.618 / >0.999 / 0.017 | 0.968 / >0.999 / 0.001 | 0.717 / >0.999 / 0.013 |
| Metapneumovirus | 0.983 / >0.999 / 0.014 | 0.654 / >0.999 / 0.016 | 0.155 / >0.999 /0.050 | <0.001 / 0.07 / 0.139 | 0.206 / >0.999 / 0.440 | 0.675 / >0.999 / 0.015 | 0.481 / >0.999 / 0.025 |
| Rhinovirus | 0.004 / 0.28/ 0.128 | 0.029 / >0.999 / 0.077 | 0.198 / >0.999 / 0.045 | <0.001 / 0.07/ 0.138 | 0.662 / >0.999 / 0.015 | 0.690 / >0.999 / 0.014 | 0.171 / >0.999 / 0.048 |
| SARS CoV2 | 0.543 / >0.999 / 0.051 | 0.502 / >0.999 / 0.024 | 0.115 / >0.999 / 0.055 | 0.530 />0.999 / 0.022 | 0.013 / 0.91 / 0.087 | 0.092 / >0.999 / 0.059 | 0.628 / >0.999 / 0.017 |
| Influenza virus | 0.493 / >0.999 / 0.054 | 0.0766 / >0.999 / 0.010 | 0.195 / >0.999 / 0.045 | 0.698 / >0.999 / 0.014 | 0.891 / >0.999 / 0.005 | <0.001 / 0.07 / 0.119 | 0.921 / >0.999 / 0.003 |
| Human coronavirus | 0.696 / >0.999 / 0.042 | 0.893 / >0.999 / 0.005 | 0.123 / >0.999 / 0.054 | 0.509 / >0.999 / 0.023 | 0.137 / >0.999 / 0.052 | 0.266 / >0.999 / 0.039 | 0.707 / >0.999 / 0.013 |
| Parainfluenza virus | 0.293 / >0.999 / 0.068 | 0.195 / >0.999 / 0.045 | 0.588 / >0.999 / 0.019 | 0.725 / >0.999 / 0.012 | 0.400 / >0.999 / 0.30 | 0.939 / >0.999 / 0.003 | 0.004 / 0.28 / 0.100 |
| Multiple infections | 0.274 / >0.999 / 0.069 | 0.018 / >0.999 / 0.083 | 0.576 / >0.999 / 0.020 | 0.034 / >0.999 / 0.074 | 0.995 / >0.999 / <0.000 | 0.889 / >0.999 / 0.005 | 0.266 / >0.999 / 0.039 |
| Negative | 0.205 / >0.999 / 0.075 | 0.461 / >0.999 / 0.026 | 0.974 />0.999 / 0.001 | <0.001 / 0.07 / 0.125 | 0.867 / >0.999 / 0.006 | 0.718 / >0.999 / 0.013 | 0.523 />0.999 / 0.22 |
| p-value / adj.p-value/Cramer’s V. SABA: short acting beta mimetics. | | | | | | | |

| **Supplementary table 4. Age specific risk for pathogen group** | | | |
| --- | --- | --- | --- |
| **0-6 months** | **OR** | **95% CI** | **p-value / p-adj.** |
| RSV | 0.829 | (0.554 – 1.241) | 0.363 / >0.999 |
| Adenovirus | - | - | - |
| Metapneumovirus | 1.380 | (0.633 – 3.009) | 0.418 / >0.999 |
| Rhinovirus | 1.298 | (0.884 – 1.905) | 0.183 / >0.999 |
| SARS CoV2 | 0.420 | (0.179 – 0.987) | **0.047** / 0.376 |
| Influenza virus | - | - | - |
| Human coronavirus | 1.021 | (0.282 – 3.699) | 0.975 / >0.999 |
| Parainfluenza virus | 1.160 | (0.500 – 2695) | 0.730 / >0.999 |
| Multiple Infections | 1.456 | (0.852 – 2.489) | 0.169 / >0.999 |
| Negative | 1.519 | (1.037 – 2.226) | **0.032** /0.256 |
|  |  |  |  |
| **6-12 months** |  |  |  |
| RSV | 1.187 | (0.751 – 1.874) | 0.463 / >0.999 |
| Adenovirus | 0.502 | (0.153 – 1.653) | 0.257 / >0.999 |
| Metapneumovirus | 1.321 | (0.567 – 2.988) | 0.581 / >0.999 |
| Rhinovirus | 0.517 | (0.357 – 0.749) | **<0.001 / 0.009** |
| SARS CoV2 | 0.809 | (0.296 – 2.215) | 0.680 / >0.999 |
| Influenza virus | - | - | - |
| Human coronavirus | 1.364 | (0.302 – 6.160) | 0.686 / >0.999 |
| Parainfluenza virus | 1.422 | (0.544 – 3.721) | 0.473 / >0.999 |
| Multiple Infections | 3.157 | (1.499 – 6.630) | **0.002 / 0.018** |
| Negative | 0.969 | (0.629 – 1.492) | 0.886 / >0.999 |
|  |  |  |  |
| **12-18 months** |  |  |  |
| RSV | 1.586 | (0.973 – 2.586) | 0.065 / 0.585 |
| Adenovirus | 0.354 | (0.114 – 1.098) | 0.072 / 0.684 |
| Metapneumovirus | 2.531 | (0.894 – 7.164) | 0.080 / 0.720 |
| Rhinovirus | 0.718 | (0.492 – 1.049) | 0.086 / 0.774 |
| SARS CoV2 | 1.077 | (0.361 – 3.213) | 0.894 / >0.999 |
| Influenza virus | - | - | - |
| Human coronavirus | 0.827 | (0.228 – 3.000) | 0.772 / >0.999 |
| Parainfluenza virus | 0.934 | (0.401 – 2.176) | 0.875 / >0.999 |
| Multiple Infections | 0.673 | (0.416 – 1.090) | 0.107 / 0.963 |
| Negative | 0.792 | (0.505 – 1.241) | 0.308 / >0.999 |
|  |  |  |  |
| **18-24 months** |  |  |  |
| RSV | 0.967 | (0.604 – 1.547) | 0.888 / >0.999 |
| Adenovirus | 1.009 | (0.221 – 4.607) | 0.991 / >0.999 |
| Metapneumovirus | 0.888 | (0.405 – 1.947) | 0.766 / >0.999 |
| Rhinovirus | 0.940 | (0.619 – 1.427) | 0.771 / >0.999 |
| SARS CoV2 | - | - | - |
| Influenza virus | - | - | - |
| Human coronavirus | 1.102 | (0.244 – 4.985) | 0.899 / >0.999 |
| Parainfluenza virus | 1.144 | (0.436 – 3.000) | 0.785 / >0.999 |
| Multiple Infections | 0.838 | (0.490 – 1.433) | 0.519 / >0.999 |
| Negative | 0.976 | (0.615 – 1.550) | 0.919 / >0.999 |
|  |  |  |  |
| **24-30 months** |  |  |  |
| RSV | 0.754 | (0.482 – 1.178) | 0.215 / >0.999 |
| Adenovirus | 1.028 | (0.225 – 4.695) | 0.971 / >0.999 |
| Metapneumovirus | 0.462 | (0.237 – 0.902) | **0.024** / 0.216 |
| Rhinovirus | 2.101 | (1.291 – 3.420) | **0.003 / 0.027** |
| SARS CoV2 | 1.992 | (0.461 – 8.604) | 0.356 / >0.999 |
| Influenza virus | - | - | - |
| Human coronavirus | 2.457 | (0.319 – 18.947) | 0.388 / >0.999 |
| Parainfluenza virus | 0.639 | (0.285 – 1.439) | 0.279 / >0.999 |
| Multiple Infections | 0.796 | (0.470 – 1.348) | 0.396 / >0.999 |
| Negative | 1.007 | (0.638 – 1.590) | 0.975 / >0.999 |
|  |  |  |  |
| **30-36 months** |  |  |  |
| RSV | 0.887 | (0.510 – 1.543) | 0.672 / >0.999 |
| Adenovirus | - | - | - |
| Metapneumovirus | 0.620 | (0.268 – 1.433) | 0.264 / >0.999 |
| Rhinovirus | 1.572 | (0.901 – 2.745) | 0.111 / 0.888 |
| SARS CoV2 | 0.741 | (0.215 – 2.548) | 0.634 / >0.999 |
| Influenza virus | - | - | - |
| Human coronavirus | 0.402 | (0.110 – 1.473) | 0.169 / >0.999 |
| Parainfluenza virus | 0.893 | (0.308 – 2.591) | 0.835 / >0.999 |
| Multiple Infections | 0.544 | (0.305 – 0.968) | **0.039** / 0.312 |
| Negative | 0.651 | (0.351 – 1.208) | 0.174 / >0.999 |

| **Supplementary table 5. Changes of pathogen risk when having siblings** | | | |
| --- | --- | --- | --- |
|  | **OR** | **95% CI** | **p-value / p-adjust** |
| RSV | 0.735 | (0.518 – 1.045) | 0.086 / 0.774 |
| Adenovirus | 0.772 | (0.250 – 2.381) | 0.653 / >0.999 |
| Metapeumovirus | 0.445 | (0.236 – 0.875) | **0.018** / 0.162 |
| Rhinovirus | 1.686 | (1.240 – 2.293) | **<0.001 / 0.009** |
| SARS CoV2 | 0.953 | (0.413 – 2.199) | 0.910 / >0.999 |
| Infulenza virus | - | - | - |
| Human coronavirus | 0.685 | (0.227 – 2.061) | 0.501 / >0.999 |
| Parainfluenza virus | 0.781 | (0.394 – 1.549) | 0.479 / >0.999 |
| Multiple Infektions | 1.035 | (0.689 – 1.555) | 0.869 / >0.999 |
| Negative | 1.013 | (0.724 – 1.417) | 0.940 / >0.999 |
